# Author Correction: Identification of fungal lignocellulose-degrading biocatalysts secreted by *Phanerochaete chrysosporium* via activity-based protein profiling

**DOI:** 10.1038/s42003-022-04290-z

**Published:** 2022-12-01

**Authors:** Christian Schmerling, Leonard Sewald, Geronimo Heilmann, Frederick Witfeld, Dominik Begerow, Kenneth Jensen, Christopher Bräsen, Farnusch Kaschani, Herman S. Overkleeft, Bettina Siebers, Markus Kaiser

**Affiliations:** 1 Molecular Enzyme Technology and Biochemistry (MEB), Environmental Microbiology and Biotechnology (EMB), Centre for Water and Environmental Research (CWE), Faculty of Chemistry, University of Duisburg-Essen, Universitätsstraße 5, 45141 Essen, Germany; 2 Department of Chemical Biology, ZMB, Faculty of Biology, University of Duisburg-Essen, Universitätsstraße 2, 45117 Essen, Germany; 3 Evolution of Plants and Fungi, Ruhr-University Bochum, Universitätsstraße 150, 44780 Bochum, Germany; 4 Novozymes, Biologiens vej 2, 2800 Kgs, Lyngby, Denmark; 5 Analytics Core Facility Essen, ZMB, Faculty of Biology, University of Duisburg-Essen, Universitätsstraße 2, 45117 Essen, Germany; 6 Department of Bio-organic Synthesis, Leiden Institute of Chemistry, Leiden University, Einsteinweg 55, 2333 CC Leiden, Netherlands; 7 Present Address: German Diabetes Center (DDZ), Leibniz Center for Diabetes Research at Heinrich Heine University Düsseldorf, Düsseldorf, Germany

**Keywords:** Hydrolases, Biocatalysis, Protein delivery

Correction to: *Communications Biology* 10.1038/s42003-022-04141-x, published online 16 November 2022.

In the original version of the Article, an incorrect additional description of panel b in Figure 1 was included. The following sentence has now been removed:

**b** Lignocellulose is a complex and recalcitrant polymer built up from cellulose, xylan (hemicellulose), and lignin. Its degradation requires the synergistic action of various different enzymes.

