# Supplementary material for: Identification of fungal lignocellulose-degrading biocatalysts secreted by Phanerochaete chrysosporium via activity-based protein profiling
Source: Commun Biol. 2022 Nov 16;5:1254. doi: 10.1038/s42003-022-04141-x (PMC9668830; doi:10.1038/s42003-022-04141-x)
Supplement: Supplementary file 2 — Supplementary Information [file 42003_2022_4141_MOESM2_ESM.pdf]

Supplementary Information

Identification of fungal lignocellulose-degrading biocatalysts secreted by *Phanerochaete chrysosporium* via activity-based protein profiling

Christian Schmerling, Leonard Sewald, Geronimo Heilmann, Frederick Witfeld, Dominik Begerow, Kenneth Jensen, Christopher Bräsen, Farnusch Kaschani, Hermen S. Overkleeft, Bettina Siebers, Markus Kaiser

Content

Supplementary Figures 3 – 8

Supplementary References 9

a

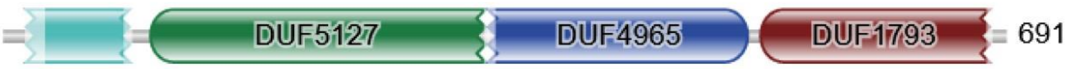

b

```
3002168                          MHSSFSLQDCPIRSLHPVTWTATPFNPASVPLAVRSPYLSAWLNQGSGTALNAD
7dks.1.A beta-glucosidase        ------------------------------------------------------
5npf.1.A Glucosylceramidase      ------------------------------------------------------
4rhh.1.A Beta-xylosidase         ------------------------------------------------------

3002168                          TSTQSTFVMTSGPIDLTVNFLSPVE---------PTDLVKQSLPFSYVTLTATS
7dks.1.A beta-glucosidase        PNSWYTYTNKDLPVQLAVKQFSPII---------PYNYKETSYPVAVFKWTAYN
5npf.1.A Glucosylceramidase      PNSWYTYTNKDLPVQLAVKQFSPII---------PYNYKETSYPVAVFKWTAYN
4rhh.1.A Beta-xylosidase         EFRVATDTWKAG--DLTLTIYSPVKAVPDPETASEEELKLALVPAVIVEMTIDN

3002168                          -VHQVQLQSQTPFGEVSDHIQQGSAFYATLAHS--GTTYQ-----TGQDTVVRA
7dks.1.A beta-glucosidase        EIVAAVMGNI---S-NDNEEWNGEYSIGVKKVPGVDISYKAKFVTTGDGSDLWH
5npf.1.A Glucosylceramidase      EIVAAVMGNI---S-NDNEEWNGEYSIGVKKVPGVDISYKAKFVTTGDGSDLWH
4rhh.1.A Beta-xylosidase         ----MRRI-DDTCPQLRGVGQGRILGIASKD----------------EGVRSAI

3002168                          GHVRDPAVEYIVAGGKTQSRSLFFWSQF----SSVGAAISSFLGDYSNALSRAK
7dks.1.A beta-glucosidase        SW-DLPIMKFGGGD--K--WYKMYTKYFGKNGKNSFAILKEALNNYQKWEKMID
5npf.1.A Glucosylceramidase      SW-DLPIMKFGGGD--K--WYKMYTKYFGKNGKNSFAILKEALNNYQKWEKMID
4rhh.1.A Beta-xylosidase         CFYRGGCVT-AGM-----DASYFYTRFF----HNIEEVGLYALEQAEVLKEQAF

3002168                          SINTSDVLMFMKEISSDGNVNTVDVIFPSWPIFLYTNPNLGKYLLLPLFEYQA-
7dks.1.A beta-glucosidase        DKRTNNMFGL-LACFDYNYYETLDVRFYGSFPLVMLWPDIEKQVMRQFADTINV
5npf.1.A Glucosylceramidase      DKRTNNMFGL-LECFDYNYYETLDVRFYGSFPLVMLWPDIEKQVMRQFADTINV
4rhh.1.A Beta-xylosidase         -----KPIWVVNGG-EYRMMNTFDLTVDQLFFELKMNPWTVKNVLDFYVERYS-

3002168                          -ALGH-------NDGNDEAMP---VEESGNMLIMTLSYVQKTG--DKSLINSYY
7dks.1.A beta-glucosidase        -IKIN-------AYD--WQNPNIWKDLNSKYVLLVYRDYVLTGKTDKEFLKYTW
5npf.1.A Glucosylceramidase      -IKIN-------AYD--WQNPNIWKDLNSKYVLLVYRDYVLTGKTDKEFLKYTW
4rhh.1.A Beta-xylosidase         SRPHYSSYELYGISGCFSHMT---HEQLVNWVLCAAVYIEQTK--DWAWRDRRI

3002168                          TDDF----AGALANQTNLAIKGIVGIKAMSQIASLAGKSSVAANYSSIAASYVT
7dks.1.A beta-glucosidase        TYDT----WSMKGTSAYCGSLWLAALKAAQEIGKVLKDNEAYIKYNEWYKIAQQ
5npf.1.A Glucosylceramidase      TYDT----WSMKGTSAYCGSLWLAALKAAQEIGKVLKDNEAYIKYNEWYKIAQQ
4rhh.1.A Beta-xylosidase         TYDSLDVSLGQARNNLYLAGKCWAAYVALEKLFRDVGKEELAALAREQAEKCAA

3002168                          -N-LFPQSVYEMHSFGVPLDTRHTYTKSDWSIWTAAIATTTAVRDLFISAVHSY
7dks.1.A beta-glucosidase        GD-ILPKDH---------------------------------------------
5npf.1.A Glucosylceramidase      GD-ILPKDH---------------------------------------------
4rhh.1.A Beta-xylosidase         -HEALRED----------------------------------------------

3002168                          VRG
7dks.1.A beta-glucosidase        ---
5npf.1.A Glucosylceramidase      ---
4rhh.1.A Beta-xylosidase         ---
```

c

```
3002168  PKNMFFNAHHSPVGAFASFTLGFP-------GKSGGLDLELGRPPRQNVY  67
   4C1P  PFNPASVPLAVR--SPYLSAWLNQGSGTALNADWPR----FWTGSILGWA  45

3002168  IGVASLSQPGMYEVLPFFEAGDDESKRYDIENPDPNPEKPQILVPFPNEM  117
   4C1P  GFIKVD---GTAYNFLGAPS----------------MPGVTFQKSV---Q  73

3002168  IQREFHVSTDTWKAG----DLTFTIYSPVKSVPNPDTAKEEDLKFALVPA  163
   4C1P  KSMTFTSTQSTFVMTSGPIDLTVNFLSPVEPTD---------LVKQSLPF  114

3002168  VIAELTIDNTK-GTSPRRAFFGFEGND------PYTSMRRIDDTCPPLRG  206
   4C1P  SYVTLTATSTDGKAHSVQLYTDISAEWVTGDNSLLANWSTAV--SSSTLV  162

3002168  VGQG-----------------RITAIVSKHSDVRSALHFS--LEDILTTP  237
   4C1P  HQVQLQSQTPFGEVSDHIQQGSAFYATLAHSGTTYQTGQDTVVRAQFVNS  212

3002168  LEENWTFGL--------GKVGALIMDTPAGM-KRTYQFAVCFYRSGYATA  278
   4C1P  GTLPNTQDTRFRAVQDAWPVFAFAHDLGSVTGTSLPVVIAGHVRDPAVE  212

3002168  -------GLDTSYFYTRFFKNIEEVGKYALDHIEALKERAFQSNQLIER-  320
   4C1P  YIVAGGKTQSRSLFFWSQFSSVGAAISSFLGDYSNALSRAKTFDAKVQGD  312

3002168  -DWLSDDQKFMMAHAIRSYYGNTQLLEQEG--------KPIWVVNEGEY-  360
   4C1P  ASKISADYASLVALSVRQAFGATEITASRNGDGSINTSDVLMFMKEISSD  362

3002168  RMMNTFDLTVDQLFFELKMNPWTVKNVLDLYVERYS---YYDRVRFPGEE  407
   4C1P  GNVNTVDVIFPSWPIFLYTNPNLGKYLLLPLFEYQATGQYPN--------  404

3002168  KEYPGGISFTHDMGVANTFSRPHYSAYELYGIDGCFSHMTHEQLVNWVLC  457
   4C1P  -----KWSVHDMGAHYPQALGHNDGNDE----------AMPVEESGNMLIM  440

3002168  AAVYIEQTKDWAWRQEKLPILEQCLESMVNRDHPDPEKRNGVMGLDSTRT  507
   4C1P  TLSYVQKTGDKSLINSYYNLLDQWTQFLITDS----------LVPA-----  476

3002168  MGGAEITTYDSLDVSLGQARNNLYLAGKCWAAYVALEKIFRDTGKEALAA  557
   4C1P  ---NQISTDDFA----GALANQTNLAIKGIVGIKAMSQIASLAGKSSVAA  519

3002168  LAGEQAEKCAATIVSY--VTEQGYIPAVMGEGNDSKIIPAIEGLVFPYFT  605
   4C1P  NYSSIAASYVTQWQGFATSKTGAHLTLSYG-NDASWGLA--YNLYGDKLL  566

3002168  NCHEALDPHGRFGEYIRALRKHLQYVLTEGICLFPDGGWKISSTSNNSWL  655
   4C1P  GLNL-----------FPQSVYEMH--SFG----------VPLDTRHTYTKS  594

3002168  SKIYLCQFIARRILGWKWDEAGAKADAAHVAWLTHPTLSVWSWSDQIIA-  704
   4C1P  DWSIWTAAIA----TTTA--VRDLFISAVHSYAADGKSAQ-PLGDWYETT  637

3002168  -GEISGSKYYPRGVTSILWLEEGK                           727
   4C1P  DGSVEGFRARPVVGGHLALVSLVI                           661
```

d

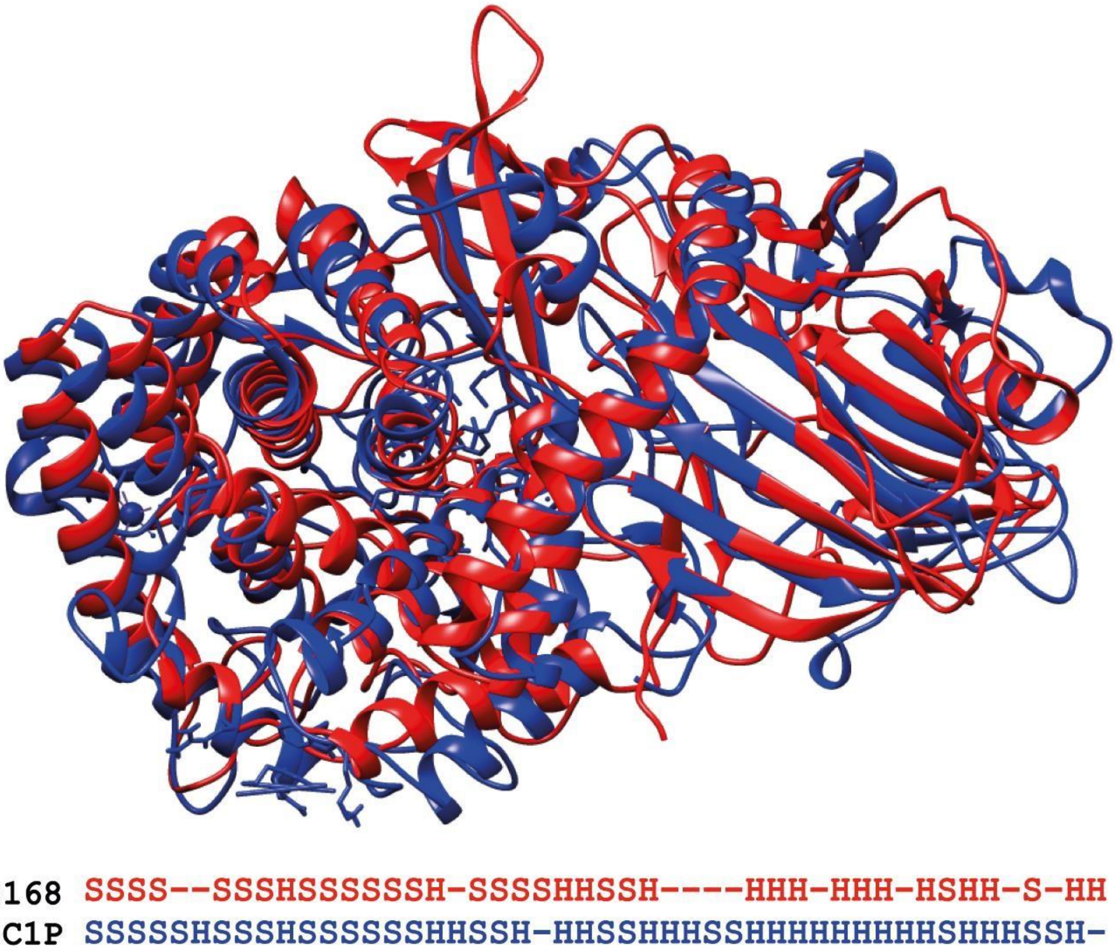

Supplementary Figure 1. Combined bioinformatics and structural analysis of the four domains of unknown function (DUF4964, DUF5127, DUF4965 and DUF1739) protein Phchr2|3002168. (**a**) The domain structure predicted by PFAM [1] and HMMER [2] shows the presence of four DUF domains (DUF4964, DUF5127, DUF4965 and DUF1739) in Phchr2|3002168. (**b**) Alignment of Phchr2|3002168 with different GH family proteins (pdb code 4RHH - *Geobacillus stearothermophilus*, pdb code 5NPF - *Thermoanaerobacterium xylanolyticum* and pdb code 7DKS - *Thermoanaerobacterium xylanolyticum* LX-11) predicted to be structurally similar by HHpred [3]. Phchr2|3002168 shows low sequence similarity to known GH52 (pdb codes 4RHH and 5NPF,17.5 % and 15.1 % sequence identity, respectively) and GH116 (pdb code 7DKS,14.1% sequence identity). Black shaded residue, same amino acid; grey shaded residue, similar amino acid. (**c**) Pairwise structural alignment by PDBeFold [4] shows that Phchr2|3002168 and pdb code 4C1P share 70% of secondary structure elements. Yellow shaded residues indicate secondary structures that are the same in the predicted structure of Phchr2|3002168 and pdb code 4C1P. (**d**) Superimposition of the Alphafold [5] predicted structure of Phchr2|3002168 with GH52 (pdb code 4C1P, 16.1 % sequence identity) and a structure-based alignment show a high similarity in predicted tertiary structure. S = betasheet; H = alpha-helix.

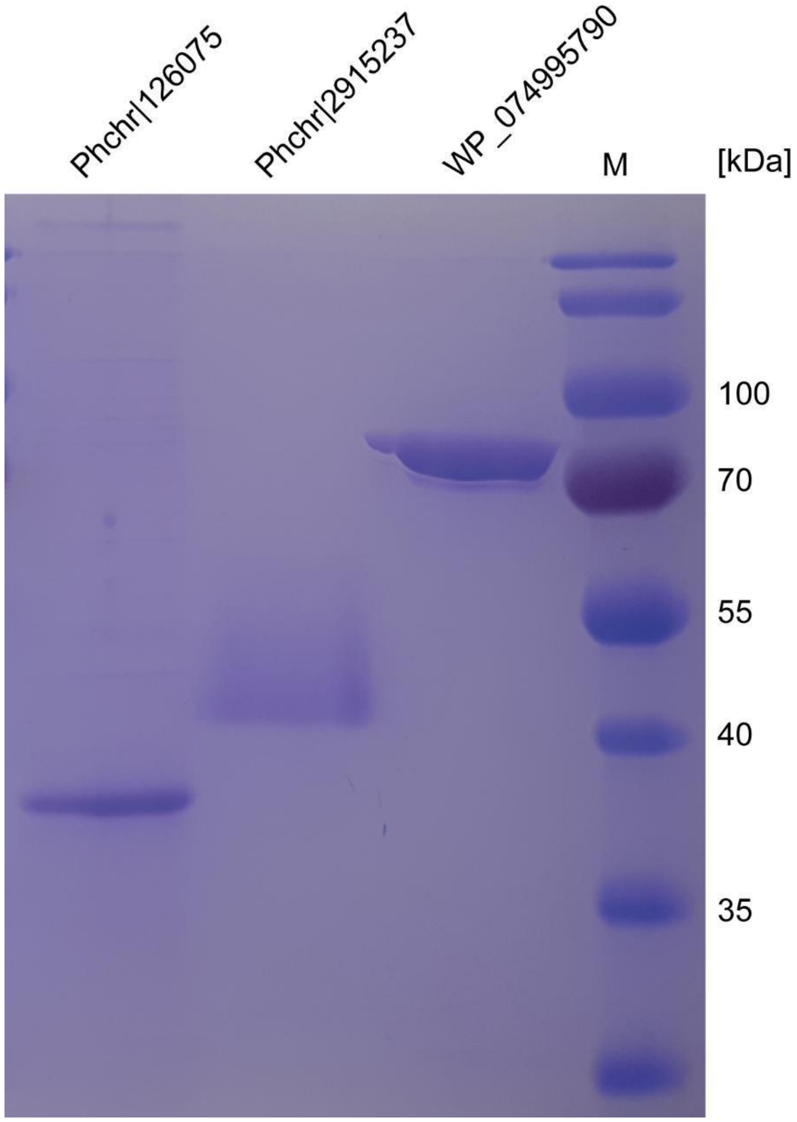

Supplementary Figure 2. Expression and purification of Phchr2|126075 *(P. chrysosporium*), Phchr2|2915237 (*P. chrysosporium*) and WP_074995790 (*S. misionensis*, close homologue of Phchr2|3002168). Phchr2|126075 was heterologously expressed in *K. lactis*. Phchr2|2915237 was expressed in *Aspergillus oryzae*. WP_074995790 was heterologously overexpressed in *E. coli* Rosetta. Proteins (2 µg) were separated via SDS-PAGE and stained via Coomassie Blue. Marker (M): prestained PageRuler™ (Thermo Fischer scientific, USA).

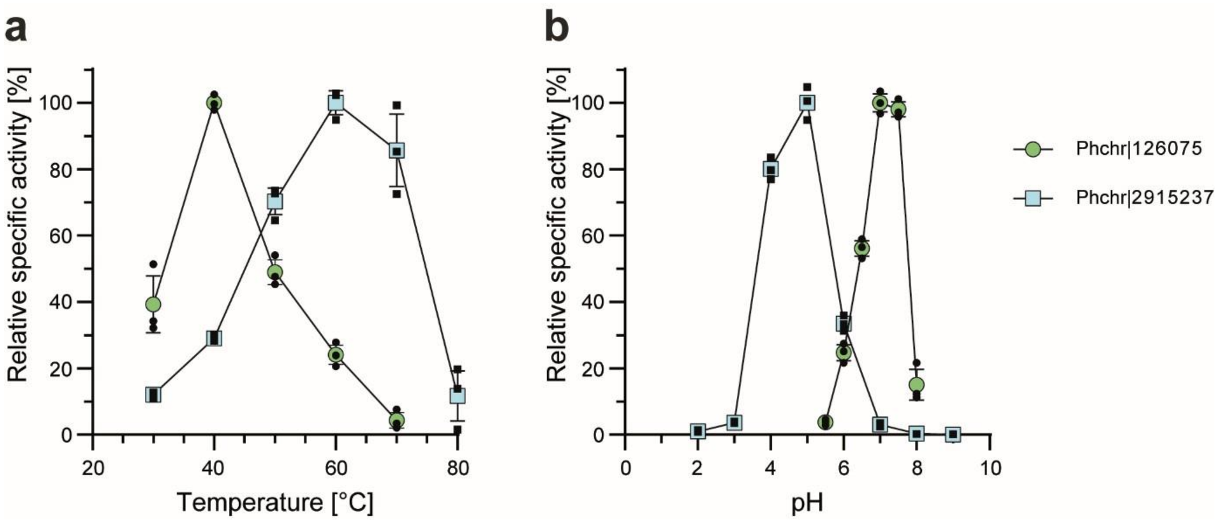

Supplementary Figure 3. Temperature and pH optimum for the heterologously expressed proteins Phchr2|126075 and Phchr2|2915237 from *P. chrysosporium*. (**a**) Temperature and (**b**) pH optimum were determined for Phchr2|126075 with *p*NP-acetate and for Phchr2|2915237 with *p*NP-Glc as substrate using a citric acid/phosphate buffer in a discontinuous assay by determining the release of *p*-nitrophenol. All activity measurements were performed in triplicate (n = 3), mean values are shown and the error bars indicate the standard deviation (SD).

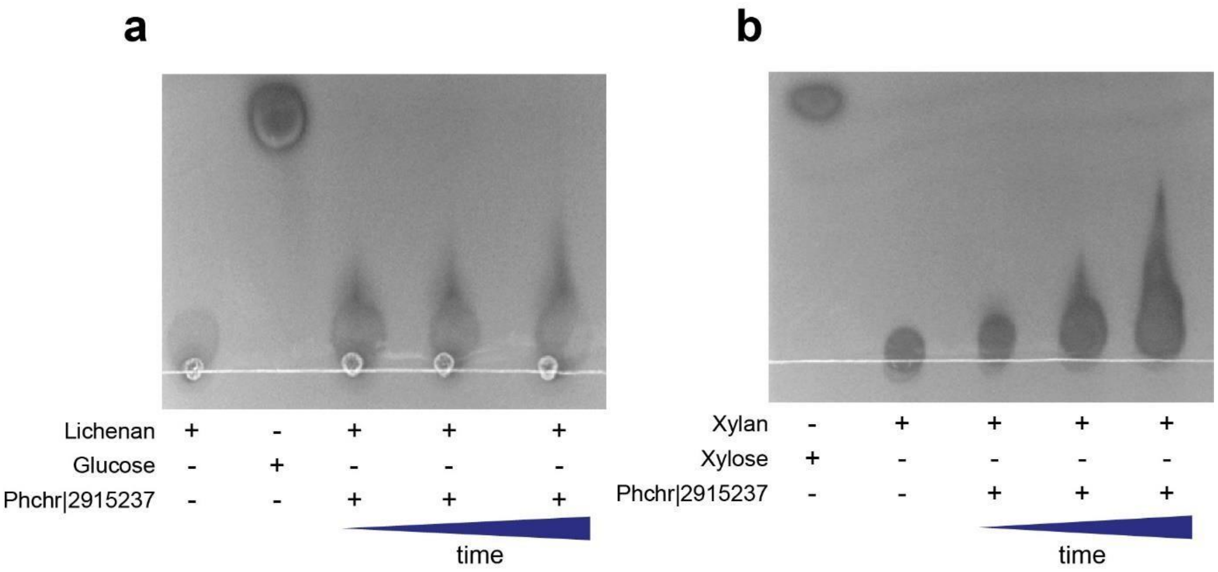

Supplemental Figure 4: Thin layer chromatography (TLC) of lichenan (**a**) and beech wood xylan (**b**) hydrolysis products formed by Phchr2|2915237. Lichenan (0.5 % (w/v)) or xylan (0.5 % (w/v)) was incubated with 10 µg of Phchr2|2915237, and the reaction products after 1, 2 and 4 hours were separated by TLC and visualized. Lichenan and glucose (a) as well as xylan and xylose (b) served as standards. Hydrolysis products and standards were separated on aluminum sheet silica gel 60/kieselguhr F254 plates using ethyl acetate, methanol and H_2O (68:23:9, v/v/v) as solvent and stained using a $KMnO_4$ solution.

Supplementary References

1 Mistry, J. *et al.* Pfam: The protein families database in 2021. *Nucleic Acids Res* **49**, D412-D419, (2021).

2 Potter, S. C. *et al.* HMMER web server: 2018 update. *Nucleic Acids Res* **46**, W200W204, (2018).

3 Gabler, F. *et al.* Protein Sequence Analysis Using the MPI Bioinformatics Toolkit. *Curr Protoc Bioinformatics* **72**, e108, (2020).

4 Krissinel, E. & Henrick, K. Secondary-structure matching (SSM), a new tool for fast protein structure alignment in three dimensions. *Acta Crystallogr D Biol Crystallogr* **60**, 2256-2268, (2004).

5 Jumper, J. *et al.* Highly accurate protein structure prediction with AlphaFold. *Nature* **596**, 583-589, (2021).
