# Supplementary material for: Identification of fungal lignocellulose-degrading biocatalysts secreted by Phanerochaete chrysosporium via activity-based protein profiling
Source: Commun Biol. 2022 Nov 16;5:1254. doi: 10.1038/s42003-022-04141-x (PMC9668830; doi:10.1038/s42003-022-04141-x)
Supplement: Supplementary file 3 — Description of Additional Supplementary Files [file 42003_2022_4141_MOESM3_ESM.pdf]

Description of Additional Supplementary Files

File name: Supplementary Data 1

Description: Full list of proteins identified by ABPP for Fig. 2b

File name: Supplementary Data 2

Description: Full list of proteins identified by ABPP for Fig. 2c

File name: Supplementary Data 3

Description: Full list of proteins identified by ABPP for Fig. 3b

File name: Supplementary Data 4

Description: Full list of proteins identified by ABPP for Fig. 3c

File name: Supplementary Data 5

Description: The source data behind all graphs in the paper
